# Effects of Early Motor Interventions on Gross Motor and Locomotor Development for Infants at-Risk of Motor Delay: A Systematic Review

**DOI:** 10.3389/fped.2022.877345

**Published:** 2022-04-28

**Authors:** Marie-Victorine Dumuids-Vernet, Joëlle Provasi, David Ian Anderson, Marianne Barbu-Roth

**Affiliations:** ^1^Integrative Neuroscience and Cognition Center, UMR 8002 CNRS - Université Paris Cité, Paris, France; ^2^CHArt laboratory (Human and Artificial Cognition), EPHE-PSL, Paris, France; ^3^Marian Wright Edelman Institute, San Francisco State University, San Francisco, France

**Keywords:** infant, motor, locomotor, early interventions, motor delay

## Abstract

**Aim:**

To systematically examine the effect of early motor interventions on motor and locomotor development in infants <1 year of age with motor developmental disability or at risk of motor delay.

**Methods:**

Pertinent literature from January 2000 to September 2021 was identified by searching the PubMed, Embase, Cochrane, Pedro and Web of Science databases. Selection criteria included interventions starting before 12 months corrected age. Methodological quality was assessed with AACPDM criteria, Mallen score and Cochrane risk of bias methodology. Evaluation procedure was performed using PRISMA protocol (PICO approach) and AMSTAR-2. This review was preregistered in PROSPERO (CRD42021286445).

**Results:**

Ten articles met the inclusion criteria; seven had moderate to strong methodological quality. The interventions included treadmill training (*n* = 3), crawling training (*n* = 1), “tummy time” (*n* = 1), physical therapy with neonatal developmental program (*n* = 1) or Bobath approach (*n* = 1), treadmill training combined with active leg movements (*n* = 2) or Bobath physiotherapy (*n* = 1). The three key characteristics of effective interventions that emerged from the review were: (1) the infants' disability or risk of delay was well-defined; (2) the protocol was standardized and easy to replicate; (3) infants were required to make active movements.

**Conclusion:**

There is an urgent need for additional high-quality studies on the effects of early motor interventions on the gross motor and locomotor development of infants with a range of disabilities or risks for delay. Suggestions for future research are outlined.

## “What This Paper Adds”

- Few early motor intervention studies exist.
- The majority of selected studies have moderate to strong methodological quality.
- Interventions were more effective when infants' disability or delay risk was well-defined.
- Interventions were more effective when the protocol was standardized.

- Interventions were more effective when infants were required to make active movements.

## Introduction

Movement plays a fundamental role in human life and has shaped human evolution. Humans move to nourish and care for themselves, to pursue prey, to escape from predators, to seek and build shelter, to communicate, to procreate, and to explore and transform the world around them. Human life would be unimaginable, and probably impossible, without the ability to move independently or to be moved by someone else. Because of its centrality in human life, movement plays a profound role in human growth and development. It sculpts the body and the brain and it provides clinicians with an accessible window into the integrity of the developing nervous system. Moreover, it enables the child to transform into an adult who understands itself and its place in the world.

Diseases, disabilities, accidents or injuries that compromise an infant's ability to move or learn new movements have enormous consequences for the infant's future quality of life. A compromised ability to move can compromise all aspects of human development and functioning, leading to a life of unfulfilled potential (1). Consequently, researchers and clinicians have advocated strongly for the provision of services and supports to infants born at risk for delayed or disrupted motor development. The prevailing consensus is that infants at risk for developmental delay should receive some type of intervention as early in life as possible [see Morgan et al., for a systematic review (2)]. Early intervention takes advantage of the heightened plasticity in the body as well as the central and peripheral nervous system that characterizes early development, thus maximizing an intervention's potential benefit (3). Even infants without disabilities or risk for developmental delay can benefit from early motor skill training. For example, daily training to step from birth promotes earlier walking acquisition (4, 5), early training to stand promotes earlier capacities to maintain a vertical posture (6) and training head control results in significantly earlier regulation of head posture (7).

Despite the prevailing consensus around the need for early intervention for infants at risk for developmental delay, the evidence in support of the efficacy of early interventions is equivocal, though it is growing stronger. Much of the evidence has been gleaned from studies of infants and young children with, or at risk for, cerebral palsy (CP), the most common childhood physical disability. The weakness in the evidence base can be attributed to several factors, including difficulties associated with diagnosing CP early in life (8, 9) and high degrees of heterogeneity in participant samples (particularly in terms of age and level of disability), the characteristics of the interventions utilized, the skills targeted for intervention, and the measures used to assess those skills. Nevertheless, researchers and clinicians have published intervention guidelines recently to promote best practices in pediatric clinical rehabilitation settings based on the available evidence (2, 10).

The current systematic review focuses on evidence for the effectiveness of motor therapeutic interventions started during the first year of life on infant gross motor development and locomotor development. Unlike previous reviews, which have not placed age restrictions on the individuals targeted for intervention or have confined the age range to children under 2 years of age, we limited our review to interventions starting during the first year of life so that we could focus on the development of the gross motor and locomotor skills that play such a vital role in the later development of motor, communication, and psychological skills. Gross motor development includes the acquisition of head control, rolling, sitting, standing up, and all skills that require the ability to maintain balance through the development of postural control and to locomote from one place to another. Postural control is vital for later skill development because it allows the child to maintain equilibrium and a particular orientation to the environment. Manipulating the environment is extremely difficult without an ability to maintain equilibrium and an orientation to it. Moreover, postural control serves as the substrate for the expression and development of all skilled activity, whether that activity is engaged in from a static base of support or a dynamically changing base of support.

The two locomotor skills that play a particularly important role in subsequent development are crawling and walking. Not only are both skills necessary for functional independence, but both have been implicated in brain and psychological development (11–13). Researchers have documented a dramatic reorganization of psychological functioning following the acquisition of crawling, characterized by changes in perceptual-motor coupling (14, 15), spatial cognition (15), memory, and social and emotional functioning (11), and they have posited an equally dramatic reorganization may follow the acquisition of walking (12, 16, 17), including in the language domain (18, 19). The pervasive effect of locomotor experience on a child's development is one of the primary reasons locomotor skill is often the prime target for therapeutic intervention for children at risk for developmental delay.

Another feature that distinguishes the current review from previous reviews is the decision to broaden the focus to infants with disabilities other than CP or risk for CP. Studies of infants with disabilities known to delay or prevent the acquisition of independent mobility, like down syndrome or myelomeningocele for example, can provide important information about the potential efficacy of locomotor interventions for infants with a range of developmental disabilities or delays. While the importance of multidisciplinary interventions is well-recognized, their efficacy is difficult to test in clinical trials. Consequently, it is important to study the impact of more focused motor interventions in order to establish a causal link with gross motor and locomotor development. Our objective is to identify which characteristics of early motor therapy have the greatest influence on gross motor and locomotor development during the first year of life and which infant characteristics moderate the efficacy of interventions. The ultimate goal is to utilize the information gleaned from the current publications to design maximally effective interventions tailored to infants with specific disabilities or at risk for developmental delay.

## Methods

This systematic review focuses on studies describing interventions designed to stimulate gross motor and/or locomotor development during the first year of life in infants with a developmental disability or at risk of motor delay.

To be included in our review, the studies had to report a training intervention that started before infants reached 1-year of age (1 year of corrected age for premature infants). Locomotor interventions could include those focused on all four limbs as well as those in which the legs might move independently, such as in early kicking, swimming, crawling, and stepping. Studies focused exclusively on upper limbs were excluded. Gross motor interventions could include studies focused on techniques designed to stimulate general gross motor development such as Bobath, Habit-il, Tummy time, etc., provided that these techniques were not combined with the stimulation of domains of functioning other than the motor domain. Accordingly, multidisciplinary interventions adding surgery, medication, interaction with parents, speech therapy, etc. were excluded. Note that, except for studies focused on locomotion, studies focused only on one specific motor skill, such as head control, sitting, standing, were excluded as we searched for practices stimulating general gross motor and/or locomotor skills. Case studies were also excluded.

As our purpose was to compare the methods used in the different training protocols, our literature search was restricted to the period between January 2000 and September 2021. January 2000 was chosen as a review starting time for two reasons: papers published before this date are often difficult to find and protocol standards have evolved making protocol comparison challenging. Our strategy for the review was to use four keywords: *Infants* AND *Motor* AND *Training* AND *Therapy*. Despite searching for training strategies focused on gross motor and/or locomotor development, we decided to keep the more general keyword Motor. This strategy allowed us to evaluate whether the motor function stimulated in each study met our selection criteria. The electronic databases used in the review were PubMed, Embase, PEDRO, Cochrane and WEB of science. We also examined the reference sections of systematic reviews to ensure we did not miss any articles, but none were included in our systematic review because they were either published before 2000 or did not meet our criteria.

**Figure 1:**
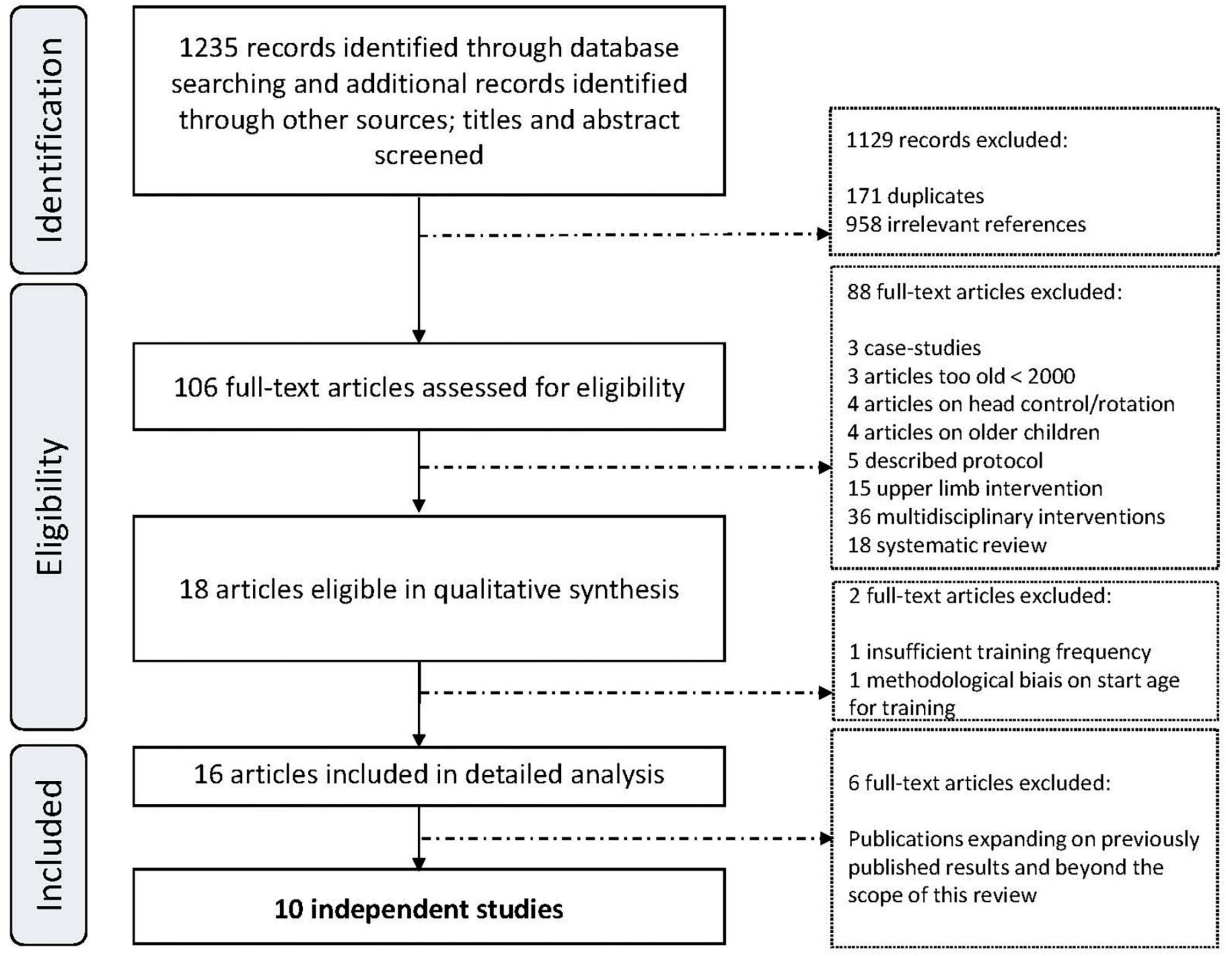
Search strategy and articles screening.

Finally, in order to avoid repetition, we selected only original papers and not subsequent papers that expanded on the original ones with supplementary outcomes but used the same cohort and protocol (see the details in Appendix S2 and in the results section). Figure 1 summarizes the research protocol and Appendix S1 provides details regarding inclusion and exclusion criteria. Methodological quality was assessed using a three-step procedure (Prisma-P statement with PICO approach; see Appendix S1 for procedure and Appendix S2 for results) with reference to the AMSTAR-2 protocol (see Appendix S3). First, we evaluated the study's methodology against the Academy for Cerebral Palsy and Developmental Medicine criteria (AACPDM) (20), including level of evidence according to Sackett et al. (21). We then determined a Mallen score (22) and finally we used the Cochrane Risk of Bias Assessment (23). Preregistration was completed on the Prospero database (CRD42021286445).

## Results

### Study Selection and Methodological Quality

Figure 1 shows the articles that were selected for evaluation. The electronic database searches identified 1,235 articles: 746 from PubMed, 223 from Embase, 30 from PEDRO, 98 from Cochrane and 138 from WEB of science. Checks for duplicates excluded 171 papers and the remaining 958 were screened based on titles and abstracts. We assessed the methodology of the remaining 106 papers.

Ninety articles were excluded because they did not meet the inclusion criteria. The remaining 16 articles were analyzed. They reported on 10 independent studies. Six additional publications (24–29) correspond to supplementary analyses of four of the 10 selected (30–33). Consequently, these six publications are not reported here because they used the same cohorts and protocols as studies that were already included. However, a brief report of their main results is provided in the results section: description of each study.

The three-step procedure revealed a high heterogeneity in methodological quality for the 10 studies. Among them, seven studies had a moderate or strong methodological quality according to the AACPDM criteria (Table 1). The Mallen scores (22) and Cochrane Risk of Bias assessment (23) are detailed in Supplementary Table S1, Table 2 and Appendix S2.

**Table 1:** Studies included in the review, methodology assessment according to the American Academy for Cerebral Palsy and Developmental Medicine (AACPDM)^a^.

**Study**	**Research design**	**Level of evidence^b^**	**AACPDM conduct questions^c^**							**Quality scores**	**Quality summary**
			**1^d^**	**2^d^**	**3**	**4^d^**	**5**	**6^d^**	**7^d^**		
Ulrich et al. (32)	RCT	II	Yes	No	Yes	No	Yes	No	Yes	4	Moderate
Ulrich et al. (33)	RT	II	Yes	Yes	Yes	No	Yes	Yes	Yes	6	Strong
Angulo-Barrosso et al. (34)	RCT	II	Yes	Yes	Yes	No	Yes	Yes	Yes	6	Strong
Campbell et al. (30)	CCT	II	Yes	No	No	Yes	No	Yes	Yes	4	Moderate
Lee and Samson (35)	Cohort study	V	Yes	No	Yes	No	No	Yes	No	3	Weak
Schlittler et al. (36)	CCT	II	Yes	No	No	No	Yes	No	No	2	Weak
Kolobe and Fagg (31)	Cohort study	V	Yes	No	Yes	Yes	Yes	No	No	4	Moderate
Wentz (37)	CCT	II	Yes	Yes	Yes	No	Yes	Yes	No	5	Moderate
Cameron et al. (38)	RCT	II	Yes	No	No	Yes	Yes	Yes	Yes	5	Strong
Ustad et al. (39)	Cohort study	V	Yes	No	No	Yes	No	No	No	2	Weak

*The order of articles presentation was made according to the type of training*.

a *Criteria for methodological quality assessment according to the AACPDM (revision 1.2) 28 with adjustments for the current study in italics*.

b *Level of evidence from Sackett et al. (21)*.

c *AACPDM conduct questions*:

*1: Were inclusion and exclusion criteria of the study population well-described and followed? Both inclusion and exclusion criteria need to be met to score “yes”*.

*2: Were the intervention and comparison condition well-described and was there adherence to the intervention assignment? Both parts of the question need to be met to score “yes.” Adherence to intervention implies that adherence is assessed in a systematic way (questionnaire, video) and that >65% of planned intervention was achieved. The cut off of 65% adherence was an arbitrary one based on common sense; it meant that about two-thirds of the intervention had been achieved*.

*3: Were the measures used clearly described, valid and reliable for measuring the outcomes of interest?*

*4: Was the outcome assessor unaware of the intervention status of the participants (i.e., was it explicitly described that the assessors were masked)?*

*5: Did the authors conduct and report appropriate statistical evaluation: that is, did they perform proper statistics and did they include a power calculation (the latter did not need to result in the demonstration of group sizes allowing for adequate power)? Both parts of the question need to be met to score “yes”*.

*6: Were dropout/loss to follow-up after start of the intervention reported and <20%? For two-group designs, was dropout balanced? Note that dropouts due to death are excluded from the dropout calculation*.

*7: Considering the potential within the study design, were appropriate methods for controlling confounding variables and limiting potential biases used? Studies with groups with n <10 at the end of the intervention—either because they started with small groups or attrition resulted in groups with fewer than 10 participants—are assigned “no,” as the small number precludes multivariable statistics to control for confounders. Methodological quality is judged—according to the AACPDM criteria—as strong (‘yes’ score on ≥six questions), moderate (score 4 or 5), or weak (score ≤3)*.

d *Criteria that address the risk of bias within studies. RCT, randomized controlled trial*.

**Table 2:** Cochrane risk of bias assessment.

**Risk of bias criteria**		**Ulrich et al. (32)**	**Ulrich et al. (33)**	**Angulo-Barrosso et al. (34)**	**Campbell et al. (30)**	**Lee and Samson (35)**	**Schlittler et al. (36)**	**Kolobe and Fagg (31)**	**Wentz (37)**	**Cameron et al. (38)**	**Ustad et al. (39)**
Selection bias	Random sequence generation	Low	Low	Low	Low	High	High	High	High	Low	High
	Allocation concealment	NF^*^	NF^*^	Low	NF^*^	NF^*^	NF^*^	High	NF^*^	NF^*^	NF^*^
Performance bias	Blinding of participants and personnel	High	High	High	Low	High	High	Low	High	Low	High
Detection bias	Blinding of outcome assessment	High	High	High	Low	High	High	Low	High	Low	Low
Attrition bias	Incomplete outcome data	High	Low	Low	High	High	High	High	Low	Low	High
Reporting bias	Selective reporting	Low	Low	Low	Low	High	High	Low	Low	Low	Low
Other bias	Other sources of bias	Low	Low	High	Low	Low	High	Low	High	High	High

Determined on the basis of information in the articles. Lack of information has been designated as “Not Found” (“NF^*^”). See Table 1 for additional information.

### General Content

#### General Characteristics of the Studies

The analysis of the articles is based on protocol type. Protocols and outcomes of the 10 studies are summarized in Table 3 and a summary of each article is provided in the results section “*Description of each study*.” In total, the 10 studies combined evaluated the effect of interventions on 219 infants (trained groups *n* = 129; control groups *n* = 90). The group sizes varied from 5 to 34 in the trained groups (median *n* = 15), and from 6 to 38 in the control groups (median *n* = 9). The Cameron et al. (38) study contributed the most infants to the review, namely 32.9% of all infants.

**Table 3:** Intervention study: training protocol characteristics (“NF” abbreviation means information was not found in research report).

**Authors**	**Population**	**Control group**	**Age start training**	**Training duration**	**Age end follow-up**	**Type of training**	**Person in charge of training**	**Frequency of training**	**Blind assessor**	**Assessment**	**Compliance evaluation**	**Main outcome**
Ulrich et al. (32)	Down syndrome	Yes	10 months	10 months (6 months and 26.5 days)	19.9 months	Treadmill	Parents	5 days per week, 8 min	Not blind	BSID II	Log book	Item 62 BSID MAW
Ulrich et al. (33)	Down syndrome	Yes	9.65–10.40 months	9.58 months // 11.71 months	19.23 months (2.80) // 21.36 (4.72)	Treadmill	Parents	5 days per week, 8 min	Not blind	BSID II	Gauge on the treadmill side	BSID II MAW Stepping
Angulo-Barrosso et al. (34)	Preterm	Yes	8.3–12.7 months	6.1 months	15.1 (3.0)	Treadmill	Parents	5 days per week, 8 min	Not blind	BSID II GMFM Ashworth scale	Gauge on the treadmill side	MAW
Campbell et al. (30)	Peri-ventricular brain injury	Yes	2 months	10 months	12 months CA	Treadmill stepping kicking	Parents	5 days per week, 8 min	Blind	AIMS GMFCS	Diary record exersises	AIMS GMFCS
Lee and Samson (35)	Myelo-meningocel	No	0–6 months	12 months	14 months	Treadmill stepping bouncing	Parents	5 days per week, 10 min	Blind	BSID II	Gauge on the treadmill side	BSID EMG MAW Stepping
Schlittler et al. (36)	At risk of NMD	Yes	6 months	7 months	12.8 months	Treadmill Physiotherapy (based on Bobath principles)	Physiotherapist	Twice a week	Blind	AIMS	NF	MAW AIMS
Kolobe and Fagg (31)	Risk of CP	Yes	4.5–6 months	3 months	7.5–9 months	Crawling	NF	Twice a week	Blind	3D analysis of movement	Videotaped	MOCS
Wentz (37)	Down syndrome	Yes	0–5 months	12 months	10 months	Tummy-time	Parents	Daily 90 min training	Blind	BSID III	Log of daily training	BSID III
Cameron et al. (38)	Preterm	Yes	Birth	6.5 months	4 months CA	Physiotherapy (neonatal developmental intervention)	Physiotherapist with parents actively involved	Weekdays	Blind	AIMS	Parental questionnaire	AIMS
Ustad et al. (39)	Brain damaged	No	5–9 months	10 months	15 months 3 weeks to 19 months CA	Physiotherapy (based on Bobath principles)	Physiotherapist	ABAB structure of 4 weeks	Not blind	GMFM 66 GMFM 88	NF	GMFM 88 GMFM 66

*The order of presentation of the articles was made according to the type of training*.

#### Infant Characteristics

Three studies included infants who had cerebral damage, including periventricular brain injury and intraventricular hemorrhage IVH (30), unilateral or bilateral cerebral palsy (CP) (39) or high suspicion of CP due to an abnormal score on the TIMP scale (31). Three studies included infants with down syndrome (32, 33, 37). Two studies included preterm infants (34, 38). One study included infants diagnosed with myelomeningocele (35) and one study included infants with a mix of inclusion criteria (36). Four studies included prerequisite assessment of the infant's developmental status before the onset of the intervention: ability to sit for 30 s (32, 33), ability to produce 10 steps on a treadmill (34) and head control (36).

#### Training Characteristics

The mean starting age was 5.6 months (SD 3.7 months) and the mean ending age was 13.8 months (SD 6.2 months). Three of the 10 studies exclusively used a treadmill for training purposes (32–34). Three other interventions mixed treadmill training with either other active leg movements such as stepping, kicking or bouncing (30, 35) or physiotherapy based on Bobath principles (36). One intervention was based on crawling movements with a supporting device (31). One intervention was based on a “tummy-time” approach (37). Finally, two studies exclusively used early physiotherapy: either a neonatal developmental program (38) or physiotherapy based on the Bobath approach (39). In six studies, parents provided the intervention (30, 32–35, 37). In two studies a physiotherapist provided the intervention (36, 39) and for one more study, physical therapists provided the intervention, but parents also actively participated in the neonatal development program by incorporating program activities into daily life (38). One study did not specify who provided the intervention (31).

#### Outcomes of the Studies

Nine of the 10 studies assessed motor development with a published developmental scale as an outcome of the intervention (30, 32–39). The last one focused on 3D analysis of movement with a Movement Observation Coding Scheme (MOCS), a scale the authors developed (40). One study used in addition to the developmental scale, the Ashworth scale to evaluate muscle tone and spasticity (34). All 10 studies assessed outcomes immediately after the end of the intervention and eight studies also evaluated the long term effects of the intervention after 12 months corrected age (30, 32–37, 39). Concerning the developmental scale used, four studies used the Bayley Scales of Infant Development II or III exclusively (32, 33, 35, 37) and two used the Alberta Infant Motor Scale exclusively (36, 38). Three mixed their approach: Angulo-Barrosso et al. used Bayley, GMFM and the Ashworth Scale (34); Campbell et al. used AIMS and GMFCS (30); Ustad et al. used GMFM 66 and 88 (39).

### Description of Each Study

#### Type, Frequency, Duration, and Effect of Early Intervention

Ulrich et al. (32) studied the effect of a treadmill training intervention on the emergence and quality of walking in 30 infants with down syndrome. Requirement for entry into the study was ability to sit for 30 s (Item 62 BSID II; mean age 10 months, SD = 2 months). Infants were randomized into either an experimental group receiving a treadmill intervention, or a control group without treadmill intervention. Both groups also had traditional physiotherapy. Treadmill training (belt speed 0.2 m/s) occurred 5 days per week, for 8 min a day, at home until walking acquisition. Parents were trained to support their infants on treadmills and completed a log book that was checked by researchers during their biweekly visits. The main study outcome was earlier occurrence of independent walking, defined as 3 independent steps (item 62 BSID II and item 42 BSID III), in infants in the experimental group, who walked 101 days earlier than those in the control group (mean 19.9 vs. 23.9 months, *p* = 0.02).

Ulrich et al. (33) explored the effects of different levels of intensity of treadmill training in infants with down syndrome. Parents were trained to support their infant on the treadmill and staff members came every other week to supervise training. The criterion to start training was the “ability to take a minimum of six supported steps in a given minute on the treadmill.” Infants were randomly assigned to one of two groups: Low Intensity (LG, *n* =1 4; 9.65 ± 1.61 months corrected age) or High Intensity training (HI, *n* = 16; 10.40 ± 2.14 months corrected age). The low intensity group received treadmill training at a fixed belt speed of 0.15 m/s. The high intensity group received treadmill training at an increasing belt speed from 0.15 to 0.30 m/s. Ankle weights were also added during HI training. Training continued until infants could walk three independent steps. The results revealed that infants assigned to HI training acquired two motor milestones earlier: “*moves forward using prewalking methods*” (item 43 BSID II) and “*raises self to standing position*” (item 52 BSID II). No significant effect was found by direct analysis of the item 62 “*walks alone*” (mean corrected age 19.23 ± 2.80 for HI group; 21.36 ± 4.72 for LI group) but a partial component analysis of the eight motor milestones implicated in locomotor development, suggested a positive effect in favor of high intensity training (*p* = 0.04).

The strengths of these two studies included the homogeneity of the groups, the standardized protocols with high frequency training sessions, specific criteria to start and end the interventions, and outcomes directly related to training (stepping performance and mean age of walking). Furthermore, clear descriptions of the protocols and results allow for replication of the studies. Ulrich et al.'s study provided a baseline control group for gait development in children with down syndrome. Ulrich et al. then set up a new trained cohort, divided into the Low Intensity Generalized (LG) and High Intensity Individualized (HI) groups, leading to the publication in 2008. A variety of dependent variables collected from both cohorts led to five subsequent publications, which are not reported in detail here to avoid repetition, but whose findings clearly showed better positive effects of High Intensity Individualized treadmill training on different outcomes even several months to 1-year post-training. This longer-term beneficial effect of HI training was reported not only on gait parameters including step length, walking cadence, stance phase and joint kinematics, but also on other parameters with HI trained subjects showing better obstacle avoidance strategies and better physical activity rate as measured by an actiwatch^1^.

An additional strength of the Ulrich et al. (33) study was the individualization of the home training. Individually tailored increases in treadmill belt speed are likely to induce better performance for each child. Regarding limitations, in Ulrich et al. (32) assessors were not blind and physiotherapy was not controlled between groups. For both Ulrich et al. (32) and (33), training data were reported but not analyzed to validate the effective training frequency and duration [log book in Ulrich et al. (32) and data from treadmill gauge in Ulrich et al. (33)].

Angulo-Barroso et al. (34) replicated Ulrich et al.'s (32) protocol with a group of 28 preterm infants with moderate risk for developmental delay. Inclusion criteria were: (1) moderate hypo/hypertonia or developmental delay when examined by a pediatrician; and (2) corrected age between 6 and 13 months, as 6 months was considered the minimum age to produce 10 steps on the treadmill (see below) and 13 months the maximum age to warrant a minimum length of treadmill training. Infants were randomly assigned to an experimental group (*n* = 15; corrected age between 8.3 and 12.7) or a control group (*n* = 13; corrected age between 6.3 and 11.0 months). At study entry, groups were homogeneous except on the Ashworth modified scale. Because infants in the control group had a higher spasticity score, the Ashworth score was included as a covariate in the analyses.

Treadmill training was performed 5 days per week, for 8 min a day, at home until walking acquisition (belt speed 0.2 m/s). Parents were trained to support their infants on treadmills and a small gauge attached to the side of the treadmill recorded minutes of treadmill use. The main outcomes were age at onset of walking (not operationally defined), BSID score, GMFM score, Ashworth score and quality of treadmill stepping. For the last outcome, two experimenters visited families monthly and recorded five 1-min treadmill stepping trials.

No group differences were found for onset of walking (experimental = 15.1 months, control = 14.6 months), BSID II or GMFM. However, regarding stepping quality, the experimental group had a higher rate of alternating stepping than the control group at 13 and 15 months corrected age, a more mature characteristics of stepping. A major strength of this study was the highly reproducible protocol it used and the clear description of its measurements and results. However, the study had several limitations. First, the control group showed higher spastic response as measured by the Ashworth modified scale. This scale has not been validated for infants at-risk of neuromotor delay and despite controlling for spasticity with the analysis of covariance, a spasticity effect could be undetected at time-t but still have influenced the later development of walking. The second limitation was that only the treadmill assessment was completed by a blind assessor. Another limitation was the wide criteria used for inclusion. For example, gestational age ranged from 23.1 to 35.6 weeks. However, degree of prematurity induces a high degree of variability in motor development as well as in the severity of brain lesions (no restriction on Intra Ventricular Hemorrhage stage). High variability at study entry on a small sample could have attenuated the effects of training. In addition, the authors noted that training should have started earlier to take advantage of higher levels of plasticity. Finally, the protocol was based on the protocol used in the Ulrich et al. (32) study. However, a protocol update was published in 2008 to increase the efficacy of the Ulrich et al. (32) training protocol. For example, Ulrich et al. (33) added ankle weights to induce more active leg movements considered beneficial for motor development. This essential aspect could have been adapted for the training of the premature infants.

Campbell et al. (30) conducted a study on the effects of early kicking exercises followed by treadmill training on motor development in preterm infants with periventricular leukomalacia (PVL) or intraventicular hemorrhage (IVH) type 3 or 4 (*n* =16). Constructed as a multi-center pilot study for a controlled clinical trial, infants were randomly assigned to a training group with home exercises starting at 2–2.5 months CA to 12 months CA, consisting of training kicking and treadmill stepping (*n* =7) or a no-training control condition (*n* = 9). Parents were in charge of the training at home under the supervision of a physical therapist visiting them once per month. Parents were asked to have their infant perform different kicking exercises 8 min per day/5 days per week from 2 to 12 months CA. Treadmill stepping exercises were added at 4 months CA until 12 months CA. Parents had to suspend the child over a portable treadmill moving at a speed from 0 to 0.6 m/s (to be increased by the parent according to the child's stepping capabilities). Therapists made monthly visits to control, explain and update the kicking and treadmill stepping exercises according to the infant's age. The Alberta Infant Motor Scale was used to assess motor development at 2-, 4-, 6-, 10-, and 12-months CA using a blinded assessor. At 12 months CA, infants were classified as normal, delayed or having cerebral palsy by a pediatric rehabilitation medicine physician. For infants with CP, a physician assigned a functional level based on the Gross Motor Function Classification System (GMFCS) categories for children before the 2nd birthday. At 12 months CA, 43% of infants in the exercise group walked alone or with one hand held vs. 11% of infants in the control group, but the difference was not statistically significant. No significant differences were found in AIMS scores either. Finally, the percentage of infants developing CP was not significantly different between the trained and control group. Regarding CP diagnosis, 42.8% of the infants in the exercise group were assigned a disability level on GMFCS compared to 33.3% in the control group (ns). To note, the frequency of training provided by the parents was lower than requested and was more likely to be 2 or 3 days per week and even lower when the infant was 6 to 12 months CA.

The strength of this article is the novel combination of kicking and treadmill exercises involving parents and starting as early as 2 months CA until 12 months CA. A major limitation was the heterogeneity in brain injuries between the experimental and control groups. Periventricular brain injury or IVH can affect the brain with different levels of severity (homolateral or bilateral lesion). A matching logic based on the brain's lesion severity should have been used to homogenize the groups at the start of the intervention. The parental compliance was lower than expected and therefore also a limitation and the actual training durations were not provided. Finally, a more sensitive developmental scale such as the TIMP might have been used instead of the AIMS to evaluate the effects of the motor training, as AIMS norms have been found to contradict medical diagnosis on several occasions [see Cameron et al. (38)] (33). We should note that one subsequent publication [see Campbell et al. (29)] (28), which is not reported in detail here to avoid repetition, analyzed the effect of kicking training alone between 2 and 4 months in the same population trained in Campbell et al. (29). However, no more positive results were reported.

Lee and Samson (35) conducted a feasibility study on the effects of early treadmill practice on infants with myelomeningocele (MMC). Twelve infants were recruited but two were excluded, one due to lack of compliance and one because the family moved away. Ten infants were included in the final analysis (mean age 2.1 ± 1.5 months). The level of spinal lesion was from L3 to L5-S2. Parents were trained to administer the treadmill stepping practice 5 days per week, 10 min per day for 12 months, starting within 6 months post-birth. Initially, treadmill training was combined with stepping and bouncing practice on a stationary surface. After 4–6 weeks of this mixed training, parents had to focus exclusively on treadmill training. Regular assessments were conducted, which included anthropometrics and motor skill acquisition using the BSID III, treadmill stepping behavior and integrity of peripheral 1a pathways with electromyography. Eight infants completed 12 consecutive months of training and 2 completed 6 months of training. Gross motor milestones appeared to be in the ranges for typically-developing infants, though walking emerged 2.2 months earlier in these trained MMC infants if compared to MMC infants described previously in the literature (41). Despite low compliance to the training protocol, an increase in alternating steps was observed. In addition, the balance between quadriceps, gastrocnemius and tibialis anterior muscles trended toward equilibrium after training and a significant increase in the ratio of quadriceps reflex responses was observed. The strength of this study was the combination of a treadmill protocol with Bayley developmental scale assessment and electromyography, demonstrating the feasibility of early treadmill training for infants with MMC. However, statistical analysis of motor milestone accomplishment was not conducted and the MMC trained group was not compared with a MMC untrained group and a typically-developing control group. The experimental group was not homogeneous in terms of birth term and age at the start of training, anthropometric data were not presented and there was no blind assessor. Despite these limitations and the bias (cf. Table 2), the results of this study are positive in showing the feasibility of such training and the improvement of motor skills, especially treadmill stepping, in this population of MMC infants.

Schlitter et al. (36) conducted an intervention for infants at risk of developmental delay. Physiotherapy based on Bobath principles was coupled with treadmill training and the effects were studied on walking acquisition and motor development. Fifteen babies were enrolled. Ten infants at risk of developmental delay were divided into 2 groups of five infants: an Experimental Group (EG; 5.8 ± 0.4 months corrected age) trained on a treadmill who also received physiotherapy based on Bobath principles; an At-Risk Control Group (RCG; 6.1 ± 0.4 months corrected age) received only physiotherapy based on the Bobath approach but did not receive treadmill training. Five infants who were not at risk for developmental delay formed a Typically-developing Control Group (TCG; 7.4 ± 0.9 corrected age), they received neither treadmill intervention nor physiotherapy. Risk criteria of the EG and RCG groups were defined according to moderate prematurity, low birth weight (<2,500 g), neonatal respiratory distress syndrome, intrauterine growth restriction, neonatal convulsions, cardiorespiratory arrest, prolonged mechanical ventilation, prolonged oxygen therapy, prolonged parenteral nutrition, fetal suffering during birth, apnea and first and second degree intraventricular and periventricular hemorrhage.

The experimental group and At-Risk control group received the same amount of physiotherapy, by a trained professional at rehabilitation centers, at least twice per week. The experimental group also received the treadmill intervention 8 min twice per week, after the physiotherapy session. Both groups started these treatments around 6 months CA, as soon as head control was acquired. All the infants were followed until walking attainment, with monthly AIMS assessments. The results did not show significant differences for age of acquisition of independent walking between the EG (12.8 months CA) and the RCG groups (13.8 months CA) or between the EG and TCG groups (12.7 months). The only difference was between the RCG and TCG groups, with At-Risk Control Group infants trained with the Bobath technique walking at 13.8 months CA, delayed compared to the Typically-developing Control Group (1.1 months; *p* < 0.05). The EG showed an increase in alternated walking steps on the treadmill between 8 to 9 months of corrected age and between 9 and 10/11/12 months CA (*p* < 0.05). No significant differences were found on AIMS scores.

The strength of this protocol was the attempt to combine an active treadmill intervention with passive/active physiotherapy to improve the effect of the intervention. The major limitation was the breadth of the definition for at-risk, which may have been too broad to permit significant results to be found in such a small cohort. The Typically-developing baseline data may not represent the typical population given the small size of the cohort and the variability in infant development. In this study, for some assessments TCG scored below the fiftieth percentile but well above on the next assessment. Other limitations were the lack of compliance evaluation, lack of descriptive data on the infant samples, lack of clarity in the description of the results and amount of physiotherapy (number and duration of physiotherapy sessions). Finally, assessors were not blind and the typically-developing control group was not assessed at 6 and 14 months.

Kolobe and Fagg (31) built a protocol based on robotic reinforcement and error-based learning of early quadrupedal locomotion/crawling. They compared the effect of crawling training with a reinforcement strategy alone or with a reinforcement strategy combined with an error-based strategy on infants with or at risk of CP (*n* = 24; TIMP for risk evaluation). A group of premature infants (*n* = 6) but at low risk of CP (defined as typically developing infants (TD) in this paper) was also compared while trained with only a reinforcement strategy to the effect of training on typically developing (TD) infants (*n* = 6). All infants in the TD group were born preterm, 60.8% of the at-risk CP infants were born preterm. Training sessions started between 4.5 and 6.5 months of age with the infant lying prone on a Self-Initiated Prone Progression Crawler (SIPPC) linked to a robotic system that allowed the infant to move forward when using appropriate crawling movements. Each session lasted 15 min, with 2 breaks of 1 min, depending on the infant's tolerance. Sessions occurred twice per week for 12 weeks at home or in child care. Arm and leg movements were blind coded from video recordings. A Movement Observation Coding Scheme (MOCS) subscale 3 was used to code the head, trunk, arm and leg movements directed toward prone propulsion. The six typically developing (TD group) premature infants and 14 of 24 at-risk infants were trained with a reinforcement learning procedure (R). The remainder of the at-risk infants (10 infants) were trained with a reinforcement and error-based learning procedure (RE) in which appropriate gestures/movements recognized as crawling attempts triggered corresponding displacements of the SIPPC. The authors distinguished two primary outcomes: locomotor goal-directed movements and the distance traveled by the SIPPC. Both were improved by the training in the TD-R group and the RE group of at-risk infants, but not in the R-group of at-risk infants. The strength of this article was the clear finding highlighting the necessity of combining reinforcement learning and error-based learning to improve crawling in the infants at-risk of CP infants while in TD premature infants a positive effect was obtained with reinforcement learning alone. Limitations included the lack a control group of infants at risk of CP without training, and lack of data on the actual training duration. It would also have been interesting to test a group of typically developing infants with reinforcement learning and error-based learning (RE). Furthermore, as none of the typically developing infants were born term, it would have been more appropriate to name this population as low risk of CP.

Wentz (37) studied the effects of a “Tummy Time” early intervention on infants with down syndrome. Nineteen infants were recruited to be trained and a retrospective study was done to construct a control group (*n* = 9; mean age = 64.0 ± 10.52 days). Intervention groups included an early start group (training starting before 3 months CA, *n* = 10; mean age = 40.9 ± 24.48 days) and a late start group (training started between 3- and 5-months CA; *n* = 9; mean age = 95.0 ± 19.24 days). Parents were asked to initiate the tummy time intervention each day until infants could independently move in and out of a sitting position. A list of tummy exercises was given to the parents and they were able to choose their own activities to engage their infants during training. A log of daily training was kept by the parents to assess compliance. The control group likely engaged in tummy-time but the authors postulated it was without additional engagement provided to the infants. Monthly motor assessments were conducted using the BSID III motor scales with the motor composite score (gross motor and fine motor score) from baseline to 12 months. A health questionnaire was given to the trained group but no health information was collected for the control group. No anthropometric differences were found between groups. Regarding the motor composite score, no differences were found between the three groups at month 1 and no differences were found between the late-start group and the control group after month 1. But the early-start group scored significantly better than the control group at month 2, 3 and 4. The early-start group scored significantly better than the late-start group at month 2 and 3. In general, an early start of the tummy time intervention had a larger effect on motor development than a late start. The strengths of this protocol included the 12 months' duration of the intervention and the follow up with the BSID as well as the simplicity of the protocol for parents, resulting in high compliance. Regarding the sample of tummy time exercises, the authors made parents aware of the risks of over stimulating spinal hyperextension and used adaptations to preserve spinal curvature, for example, by using towels under the arms to avoid neck hyperextension in the prone position. Regarding limitations, no data were collected relating to the infants' medical histories or relating to potential physical therapy engaged in by the infants. Further, a blinded assessor was not used and tummy time data for the control group were not collected. Finally, the representativeness of the sample is questionable because the authors excluded infants who were prone sleepers. It may have been better to include these infants but ensure there was an equal number in each group.

Cameron et al. (38) studied effects of an early *Neonatal Developmental Intervention* for very preterm, very low birthweight (VLBW) infants. Seventy-two infants born very preterm (GA <32 weeks) with VLBW (BW <1,500 g) were randomly assigned to a non-treatment (NT) (*n* = 38; mean GA =2 8.7 ± 2.4) or treatment (T) (*n* = 34; mean GA = 29.6 ± 2.0) group. A control group of typically-developing infants born at term (*n* = 14) was also recruited at 4 months of age. The treatment group received the developmental training from birth until 4 months CA. The training was provided 5 days a week during the infant's neonatal stay at the hospital and on a needs- and problem-oriented basis thereafter until 4 months corrected age. Training occurred on weekdays with a maximum of 10 min duration. The neonatal developmental program promotes musculo-skeletal symmetry in relation to the median axis, cervical and lumbar rotation and also movement experience. It encourages head symmetry by using visual tracking on both sides and hand to mouth contact. Strengthening neck, trunk and leg flexors is also included as much as the prevention of contractures. Therapy was given by physiotherapists and also by parents, who were trained to reproduce the specific techniques on a daily basis. Whether delivered by the therapist or the parents, the program was adapted to the infant's age and to the infant's progress. Weekly classification using the Longitudinal Assessment of Preterm Infants (LAPI) allowed for a fixed frequency of therapist intervention after discharge, with sessions structured as 40 min for assessment and physiotherapy, and 20 min for parental instruction. A parent questionnaire was used for parental compliance and a blinded assessor assessed all infants with the Alberta Infant Motor Scale at 4 months CA at the hospital. No significant effect of the physiotherapy was revealed on the treatment group's motor performance on the AIMS. But in the treatment group, families with high levels of parental compliance (38%) had better scores on the AIMS than those with lower parental compliance (*p* = 0.05). However, the use of the AIMS is problematic in this study. Theoretically, AIMS scoring is related to abnormal motor development predictability when the score is below the 10th percentile. Despite this, in the preterm treatment group, no infants had abnormal motor development at 4 months CA, and in the preterm no-treatment group, 16% of the infants had abnormal motor development compared to 14% in the typical control group. The differences between the three groups did not reach significance (p=0.09). The second problem was the lack of correspondence between the AIMS scores at 4 months CA and the development of CP: five infants were detected by AIMS with potential CP, although only two of the five infants received an official CP diagnosis. Finally, 12 preterm infants were medically diagnosed with CP at 18 months corrected age. The strength of this protocol was the high number of subjects and early stage of the training, starting in the NICU at birth, which capitalized on the window of highest developmental plasticity. The limitations concerned the use of AIMS, but also recruitment and compliance of the parents. Even if the differences were not significant, the trained and control groups were not homogeneous with 53% of the treatment group having normal brain development compared to 36% in the no-treatment group; and for grade III/IV/PVL there was 9% in the treatment group vs. 18% in the no-treatment group. Furthermore, infants in the experimental group had significantly more advanced terms and higher birthweights than infants in the control group. Finally, parental compliance was a limitation, with 38% of the sample showing low compliance in the treatment group. All these limitations could explain the inconclusive results obtained in this study.

Ustad et al. (39) studied the effect of physiotherapy training alternating between physiotherapy as usual (period A, varied amount of session) or intensive physiotherapy (period B, daily session) on five infants aged from 6 (mean 30.2 ± 6.1 weeks) to 12 months (mean 54.2 ± 6.1 weeks), diagnosed with or with a strong probability of having CP and classified according to the GMFCS. A single-subject, multiple-baseline, withdrawal design was used (ABABA). Therapy as usual (A) was defined as sessions once per week or once every second week, during 8 weeks. Intensive physiotherapy sessions (B) were defined as 1-hourly intervention, 5 days per week, during 4 weeks. The sessions were adapted to the child's endurance with durations varying from 40 to 60 minutes and a maximum of 19 sessions during the period (B). The intervention was designed on the basis of current principles of the Bobath approach and by motor learning principles: a total of 3 to 8 goals were set per child to stimulate the functional areas of locomotion, sitting and bilateral hand function. At least one parent was present at each session, carrying and handling skills were incorporated into the intervention. The children were initially assessed using the GMFM 66 & 88 and then the same GMFM was assessed every fourth week by a blinded assessor.

All children had significant improvements in GMFM scores from the baseline period, but the increases in scores came during both periods A and B. Consequently, the effect of intensive vs. usual physiotherapy was inconclusive. The strengths of the protocol included the specific tailoring of the intervention to each infant with a specific therapeutic protocol and the high frequency of therapy sessions during a period in development characterized by high plasticity.

The sample size was small, but each subject served as his or her own control, which can be considered a strength, especially since the protocols were adapted to the subject. The statistical analyses are therefore related to the progress of each subject and there is no control group. This last point represents both a strength, because it solves the ethical question of the control group which does not receive the innovative intervention, but also a limitation because from a statistical point of view, it reduces the power of the intervention. The high degree of variability in the physiotherapy sessions was another limitation. No information was given about the real training duration (no log book or record). For period A, sessions were once per week or once every 2 weeks, without specific duration. For period B, daily sessions could last from 40 to 60 min, which represents 800 min (13 h) variability for the two periods of 8 weeks. Furthermore, the authors used the GMFM test, which is known to be insensitive to detect changes over small-time intervals. Although it is a standardized tool, Kolobe et al. (42) note that it should be used for regular examinations with a minimum test interval of 3 months to observe motor development.

## Discussion

The purpose of this review was to determine the effectiveness of motor therapeutic interventions started during the first year of life on gross motor and/or locomotor development in infants with a range of developmental disabilities or who were at risk for developmental delay. The rationale for focusing on the development of gross motor skills and locomotion was based on the central contribution these skills make to the emergence and expression of later developing motor and psychological skills. The rationale for including infants with a range of disabilities and risk factors was based on the desire to examine the robustness of the various interventions that have been implemented. Our objective was to identify which characteristics of early motor therapy have the greatest influence on gross motor and locomotor development and which targeted population and protocol characteristics moderate the efficacy of interventions. The ultimate goal was to allow researchers and clinicians to utilize the information gleaned from the current publications to design maximally effective interventions tailored to infants with specific disabilities or at risk for developmental delay.

Over a period of 21 years (2000–2021), 10 independent studies met our inclusion criteria (30–34, 37, 38). The majority of studies were small randomized controlled trials, with a total sample equalling 219 infants; seven of 10 studies were designed either totally or partially to promote the acquisition of independent locomotion. The three other studies were designed to stimulate more general gross motor development. Only three studies were considered strong in terms of methodological quality (33, 34, 38); the others were rated as moderate or weak in methodological quality, primarily because they did not use blinded assessors, had low compliance with the intervention, or did not report in sufficient detail all of the information necessary to ensure the study's replication (see Table 1). The risk of bias varied considerably, ranging from low to high. The participant inclusion criteria were not always well-defined. Despite all the limitations in the studies, our review reveals that early motor interventions targeting gross motor and/or locomotor development can be effective. Four of seven studies mainly targeted on treadmill or crawling training showed positive effects on locomotor/motor skills (31–33, 35) and one (37) of three general gross motor interventions showed positive effects on motor development. What aspects of these different interventions could explain why some were more effective than others?

Comparing the different studies' approaches proved difficult because there was so much heterogeneity in the population samples, the protocols and the outcomes assessed. This problem was exacerbated in studies that used manual therapy exclusively or used mixed strategies because it was difficult to determine the exact role played by the therapist vs. the protocol. Among other challenges, the therapist is torn between standardizing the protocol to prove its effectiveness or adapting the therapy to each patient and his or her unique rate of progression. The results of studies that utilized specific locomotor training of treadmill stepping/ kicking/bouncing/stepping (30, 32–35) or crawling with a powered mobility device (31) were easier to interpret because the bias from the experimenter/parent's participation was minimized. Despite the multiple approaches used in the interventions, we can highlight the main characteristics of the different studies and the principal factors leading to significant or inconclusive results.

### Main Characteristics of the Studies

#### Population

The targeted trained population ranged from having relatively well-defined motor risk [five studies with three on Down Syndrome, one on myelomeningocele and one on infants at high risk of CP (31–33, 35, 37)] to populations with less-defined motor risk [five studies on preterm, infants with brain lesions or at risk of NMD (30, 34, 36, 38, 39)]. Interestingly, the five studies focused on populations with well-defined motor risk reported more positive results than the studies performed on populations whose motor risks were less precisely defined.

#### Intervention

The starting age for the interventions varied from birth at the NICU or at the maternity to 10 months corrected age. The frequency of training sessions was somewhat similar, ranging between 2 and 7 sessions per week. This is in accordance with reported positive effects of shorter more frequent sessions compared to longer and less frequent sessions (2). Except for the 3 months of crawling training on a crawling robot by Kolobe and Fagg (30), all interventions were of long duration, ranging from 6 to 12 months. Consequently, the interventions selected in this review not only start very early but showed relatively high training frequencies. Intensity was another factor that enhanced the efficacy of the interventions (43). This was illustrated clearly in Ulrich et al. study where the more intense the treadmill training, the more effective it was at accelerating the acquisition of independent walking (33).

Interestingly, all studies used interventions that were largely active, that is maximizing the movements generated by the infant himself/herself. This in accordance with the literature showing that active generated mobility is more effective than passive mobility for infant development (11, 42, 44). For example, two studies with positive results used an active stimulation of prone quadrupedal movements mostly self-generated by the infant (31, 37). Other studies used interventions that were largely active, though not entirely active, with generally positive results. For example, six studies used treadmill training in which parents supported their infants on top of a moving treadmill belt (30, 32–36). This type of training has passive and active components. The parents provide postural support and the treadmill initiates each step, though active muscular contractions are required to complete the step and support a percentage of body weight. To accentuate the active component of treadmill training, Ulrich et al. added weights to the infants' ankles (32). Five studies used other protocols with various active and passive components. Three studies combined treadmill training with other stimulation, including an active kicking training with a mobile (30), stepping and bouncing (35) or physiotherapy based on the Bobath approach (36). Finally, two studies used neurodevelopmental (38) and Bobath-based physiotherapy (39), with both types of physiotherapy using active and passive components. While it is difficult to conclude what type of training was more successful than another, we observed that in general more standardized protocols including treadmill, crawling or tummy time stimulations were more successful in achieving significant results. However, this is not to neglect other protocols using physiotherapy or mixed approaches as discussed further in the section on recommendations.

#### Choice of the Developmental Assessments

Although all studies focused on assessing gross motor development, they used a variety of assessment instruments in addition to the age of walking onset and/or gait characteristics.

Five studies used the Bayley scale (32–35, 37). This is a standardized test that assesses the child's development with a minimum interval of 2 weeks, which allows for very close follow-up. This scale is normed to typical children which provides a baseline and has a standardized scoring system to compare findings across studies. Ustad et al. (39) used the Gross Motor Function Measurement (GMFM) scale every 4 weeks to assess motor development, even though others have recommended having intervals of at least 3 months between assessments with the GMFM (45).

Three studies used the Alberta Infant Motor Scale. One study analyzed the effect of training on the AIMS score but not all data were reported (36), one did not find significant results when the AIMS scale was used but protocol compliance was lower than expected (30) and one study did not find congruence between the AIMS score and a future diagnosis of cerebral palsy (38). In fact, Cameron et al. (33) found a lower detection rate of abnormal motor development at 4 months CA in preterm infants using the AIMS and the AIMS detected only two infants among the 12 infants ultimately diagnosed with CP by a medical practitioner (i.e., 17%), suggesting the AIMS may need to be revised for developmental studies of preterm infants (46, 47) or supplemented with other assessments like the TIMP (38). Finally, one study used the Gross Motor Function Classification System (GMFCS) before 2 years corrected age for infants classified as having cerebral palsy at 1 year corrected age (30). However, the evolution of brain lesions is unstable during the first years of life suggesting postponement of the GMFCS until later is warranted (30, 48–50).

### Factors Leading to More or Less Effective Interventions

#### Population

The studies highlight some of the principles that underlie effective interventions. We already highlighted the importance of the choice of the population targeted for the intervention. Interventions were most likely to be efficacious when the selected population was homogenous in their pathology or developmental risk. Only five studies selected subjects with similar pathologies, including down syndrome (32, 33, 37), myelomeningocele (35) or a strong probability of having CP (31). The other five studies had groups of subjects with variable pathologies, including wide variations in degree of prematurity (34, 36, 38) or brain damage with different levels of severity (30, 39). When a small number of subjects is available, it is important to minimize the degree of heterogeneity in the sample so that comparisons can be made across intervention protocols or between intervention and control groups. Interestingly, five (31–33, 35, 37) studies using well-defined populations reported significant results while five (30, 34, 36, 38, 39) studies using less defined populations were inconclusive.

#### Compliance

Adherence and compliance are also essential aspects of any successful intervention, regardless of the protocol implemented (36, 38, 39). A low compliance is reported as having negative effects on the results of the intervention (38). In this regard, we noted that adherence to the intervention protocol was not always well-monitored. Two studies did not report adherence (36, 39), three studies did not report actual training duration (30–32) and in two studies 65% of participants did not complete the desired level of training [AACPDM criteria—(35, 38)]. In only three studies, participants completed more than 65% of the training protocol (33, 34, 37).

#### Type of Intervention

The type of intervention and its replicability is also important for the success of a protocol. Using physical therapy exclusively (38, 39) with active and passive techniques has the advantage of stimulating the whole body and exploits the potential to adapt the therapy to the unique needs of the individual and his or her rate of progress. This potentiates the effects of therapy for each child but can negatively affect group analyses unless a within-subject analysis is conducted and each subject is treated as his or her own control (39). However, according to Cameron et al., within-subject comparisons are difficult to interpret (33). Another drawback of physical therapy is a risk of possible bias from the therapist and the application of these complex protocols renders their replicability more difficult. All of these different limitations could explain the inconclusive results of both exclusive physical therapy interventions selected in this review (38, 39).

More standardized interventions seem to get more positive results. This is the case for quadrupedal training on a device, a procedure that is promising because it involves full active participation of the child's whole body and promotes the coordination of arms and legs, which is crucial for future walking, while it also removes the potential bias from an experimenter (31). The treadmill training protocol is also a well-standardized intervention, even if there is still a potential bias from the experimenter. In contrast to quadrupedal interventions, treadmill training has the disadvantage of not engaging the whole body, especially the arms because the experimenter must support the child under the armpits, blocking arm movement. This a drawback compared to the crawling training since the arms are important for walking (51, 52). Nevertheless, most of the treadmill training studies selected in this review had clear positive effects on the acquisition of independent walking in at-risk populations. Remarkably, this effect was replicated in different populations including Down Syndrome (32, 33) and Myelomeningocele (35).

Finally, several studies combined mixed protocols; mainly by combing treadmill training with another intervention such as kicking on a mobile (30), bouncing and stepping on a static surface (35) or physical therapy based on Bobath principles (36). Combining treadmill (or crawling) training and other therapies should increase the odds of achieving significant improvements in infant motor/locomotor development. This is especially the case for protocols exploiting the benefits of standardized active training and the benefits of adaptive manual therapy, including passive and active mobilization techniques such as those based on Bobath principles. However, even if these approaches are promising, the complexity of the interventions can compromise statistical power. This is perhaps why we observed inconclusive results in some of these studies (30, 36).

### Recommendations for Research and Best Practice

Our recommendations for future research and clinical practice are organized relative to the design of interventions, the choice of assessments and the descriptions that need to be provided in published reports (see Appendix S4).

#### Choice of the Population and Inclusion Criteria Definition

Researchers should precisely define the target population for motor training in terms of age, pathology/disability, socio economic status, anthropomorphic data, etc.: too much heterogeneity in these characteristics can severely compromise the results of the training. If the target population is considered at-risk, the degree of risk and how it is assessed need to be defined and specified. The definition of risk for motor impairment or delay should take the child's birth and clinical history into consideration. Researchers should consider using Prechtl's General Movements assessment (53) or/and Amiel-Tison's Neurological Assessment (54) or/and neuroimaging [MRI/TFU (9, 55)] to determine infants at-risk and present data if available (56).

Half of the publications defined their population as having *premature birth, brain damage, risk of cerebral palsy* or *at-risk of neuromotor disorder*. These criteria can be further divided into several sub-categories. For example, prematurity could be defined by subcategories of gestational age (*24–28 GA, 28–32 GA, 32–34 GA, and 34–37 GA*) and/or birthweight (*extremely low birthweight, very low birthweight and low birthweight*), values that should also be homogenized between trained and control groups. The same recommendation is appropriate for brain lesions, which should be paired between trained and control groups, with regards to lesion location and whether the lesion is homolateral or bilateral. The diagnosis of cerebral palsy or neuromotor disorder is even more problematic in pediatric rehabilitation, because neurological signs can be variable and unstable across the first 24 months of life. For example, if cerebral palsy is suspected, clinicians typically wait 4 years to confirm the diagnosis (57). Five studies ended their follow-up at or before 1 year corrected age (30, 31, 35, 37, 38). Intervention follow-ups were generally too short to ensure that the at-risk samples actually included infants with cerebral palsy. Consequently, we recommend extending the longitudinal follow-up of trained children to at least 3 or 4 years of age when they are initially at risk for CP and to use tools that can diagnose CP earlier such as Prechtl's General Movements assessment (53) or/and Amiel-Tison's Neurological Assessment (58, 59).

#### Necessity of Control Groups

Studies should include control groups in addition to intervention groups. The control group should be comparable to the intervention group in terms of birth history, clinical history (brain damage), morphological characteristics, socio economic status and motor skill and baseline intervention (31). In the case of infants with brain damage, control group and experimental group participants should be matched on the basis of their neuroimaging results. To ensure comparability between intervention and control groups, randomized allocation of participants to groups is recommended. However, when the randomization is not feasible, for example to ensure the greatest homogeneity between groups in terms of weight, age, socio economic status, brain lesions etc. a pseudo-random allocation might be possible where paired subjects presenting similar major characteristics (brain insult, clinical history or socioeconomic status) are randomly allocated. Where possible, a group of typically-developing infants, matched with the other infants on as many characteristics as possible, should also be used for comparison purposes. Note that a typically-developing control group cannot comprise premature infants because prematurity is a risk for developmental delay. Group sizes should be large enough to ensure adequate statistical power, potentially requiring studies that use the increasingly common multi-center approach.

#### Necessity of Testing the Validity of a Protocol on Different at-Risk Populations

As mentioned in the selection process of this review, we sought interventions on populations other than the usually-targeted children at-risk of CP. As an example, here we reported on two studies focused on infants with down syndrome and one study on infants with myelomeningocele using the same treadmill training protocol, with both studies showing improvements in motor development and the acquisition of walking in their infants. These results not only confirm that treadmill training is a promising strategy for different populations at risk of motor/locomotor delay, but highlight how important it is to replicate positive findings, especially when the cohorts are small due to the enormous work demanded by such interventions.

#### Training Choice in Function of Objectives

Motor training should start as early as possible to take advantage of the window of highest plasticity in the body and nervous system. The frequency and duration of training should be as high as possible. Daily training will likely involve parents delivering the intervention, whereas weekly training can be conducted by a therapist. The best solution is probably to combine daily training by the parents and a weekly or bi-weekly visit from the therapist to train the infant. This allows the therapist to verify the compliance with the training and to decrease the burden of this training on the parents. Adaptation of training needs to be considered relative to the planned analyses. If duration is adapted to the needs of the participant, then analyses of effects of training can be done in relation to time 0 and participants can be compared with respect to progress attained after a certain amount of training. If duration is fixed, comparisons among participants are possible at any time during the follow up. The training modality should be standardized across all individuals within the training group to allow valid conclusions about the efficacy of the training. In any case, the number of sessions and the duration of each session must be recorded, preferably using automated technology that places minimal burdens on parents and cannot be tampered with. The duration of the follow up should be determined relative to the objectives of the training.

Where possible, researchers and clinicians should use an ICF-CY^2^ intervention design. In such designs, the infant's ability to act on its environment with autonomy serves as an important reward and reinforcer of behavior. Active movements expressed by the infant have a more powerful effect on perceptual and motor development than passive movements imposed on the infant (11, 60, 61). Active production of movement can be likened to operant conditioning in which the child's production of movement is positively reinforced, leading in turn to heightened voluntary control over movement. Active movements are retained better than passive movements (62).

Regarding the type of intervention, we strongly urge researchers and clinicians to use training interventions that involve the whole body when gross motor development is the primary outcome focus of the intervention. Crawling is an ideal activity to train because it checks multiple boxes in terms of the recommendations we have made so far. Crawling can be performed from birth, it is under the active control of the infant and therefore requires no additional support from an experimenter/parent, it can be done daily, it involves the head, trunk and all four limbs, it can be enhanced by adding sensory information to the training environment (e.g., optic flow, maternal odor, maternal voice), it requires minimal equipment, and it is easy to quantify and track (63, 64). Moreover, crawling naturally adapts to the infants' developmental status—they crawl further and faster as they get stronger and more coordinated. Early aquatic interventions also warrant consideration because they too check multiple boxes in terms of the recommendations we have made so far. The buoyancy provided by the water simulates the aqueous environment experienced by the fetus and permits the control of many movements that are difficult for very young infants to express on land. Moreover, early aquatic therapy can be delivered daily in a bathtub using assistive devices that are cheap and accessible (65). If the acquisition of erect locomotion is considered the final goal of motor development in the first year of life, the treadmill stimulation has proved to be effective. However, a treadmill is difficult to use from birth, it requires the additional support from an experimenter/parent and does not stimulate the arms and trunk, which are important for future walking. So, we recommend using crawling or swimming for early intervention and treadmill stepping for later intervention.

Finally, we recommend adding sensory information to the training environment (as simple as encouragement from the parents) in order to improve the motor responsiveness in infants.

Although interventions that encourage active whole-body movements are preferred, we do not want to downplay the potential positive effects of interventions focused on joint mobilization and muscle relaxation or myotensive techniques that are in line with the recommendations of good practices in the field of early rehabilitation (10). Even if manual therapies using those techniques, like osteopathy, need better evidence for their efficacy, we recommend using them concurrently with motor interventions from birth onward, if possible, because they can prevent damage to the body that results from the combination of atypical neuromotor and physiological development.

The last consideration is whether to use a multidisciplinary or specific intervention protocol. Multidisciplinary intervention approaches can induce specific changes in motor functions but they cannot reveal which component(s) of the intervention were responsible for the changes. Consequently, we recommend approaches with a higher degree of specificity if the researcher/clinician is interested in determining what causal ingredients of the intervention were associated with positive effects. Finally, the motor training must be well-tolerated by the infant's family; if not, compliance is likely to be low.

#### Collection of Dependent Variables

Standardized assessment tools are necessary to permit replication of findings and comparison with other studies. These tools must be concordant with the training objectives. In the case of motor training and its effects on motor/locomotor development, adapted scales and tools to evaluate these skills are needed according to the test frequency and age of the infant/child. We also recommend cross referencing the data collection by using several assessment tools: parental questionnaires could be used in addition to standardized developmental scales (Bayley Scales of Infant Development, Test of Infant Motor Performance etc.). Different tests could also be used at different ages or for different purposes. For example, the Test of Infant Motor Performance (TIMP) would be more appropriate for early evaluation (as soon as 34 weeks GA) for infants at risk of CP and could be supplemented later with the BSDIII (as soon as 2 months CA) for motor and locomotor development.

The quality of locomotor development should also be assessed with specific tools (EMGs, stepping or crawling patterns, gait characteristics) rather than assessing only the age of acquisition of walking, a measure that is not very reliable, especially in small cohorts, as high degrees of variability in the sample can attenuate group differences. In place of the age of walking, it would be more appropriate to assess an item related to walking skill on a validated scale at a time-T, and then test the percentage of successful infants between groups using chi-square in order to increase the chances of demonstrating an effect of the intervention [BSID-II, item 62; Ulrich et al. (32)] (31). Assessments should be conducted at least two times—at baseline and immediately after the end of training, and if possible, several times after some delay relative to the end of training to determine whether any changes were permanent. Finally, all assessments should be conducted by an assessor blind to the participants' group and preferably also to the objectives of the study.

#### Avoid Reporting Bias

Detailed descriptions need to be provided of the participant's characteristics (e.g., morphological) and histories (birth and clinical), the features of the experimental and control interventions, the frequency and duration of the training sessions, the location of the intervention, the people who delivered the intervention, the infants' behavioral state, compliance with the intervention, attrition and reasons participants dropped out, what assessments were used and when they were used, and whether the assessors were blinded to the participants' group assignment and the objectives of the study. The instructions given to parents should also be provided. When the age at which specific motor skills are acquired is assessed, clear definitions of the skill need to be provided, e.g., sitting without support on a firm surface for 30 s, or three independent steps without falling. The infants' behavioral state (66) during training should also be recorded and reported. The instructions given to parents should be provided, preferably via copies of written instructions. For the main outcomes, all results should be reported to avoid the bias associated with selective reporting (23). One characteristic that is often missing in scientific studies is the socioeconomic status of the participants. We strongly urge researchers to report these characteristics. Socioeconomic status is important because participants vary in their potential to access therapeutic interventions based on their socioeconomic status and the effectiveness of interventions can vary according to the participant's socioeconomic status (67).

### Strengths and Limitations of the Review

The most obvious limitation of the review is the decision to restrict the inclusion of studies to those utilizing motor interventions that targeted gross motor and locomotor development and that started during the infants' first year of life. Our review is therefore narrower than similar reviews about the effectiveness of interventions for older children with developmental disabilities or at risk for developmental delay.

Both of these restrictions were deliberate. Focusing on gross motor interventions permitted a better understanding of the specific effects of these interventions and focusing on studies that started before 1 year coincided with the period of maximal plasticity of the body and nervous system, such that the interventions were most likely to be successful. In addition, gross motor development is a potential catalyst for developmental changes in perceptual, cognitive, social, and emotional domains of functioning.

In contrast, the selection criteria led us to consider protocols targeting diverse populations. As we did not restrict our keywords to one type of pathology, our review covers heterogeneous populations suffering from different developmental disorders or presenting risks of developmental delay (Down syndrome, myelomeningocele, prematurity, cerebral palsy, etc.). This method of article selection allowed us to compare the robustness of the interventions during the first year of life. However, the heterogeneity of the participants' characteristics could be considered a limitation.

## Conclusion

The systematic review enables clinicians and researchers to know what interventions have been conducted within the last 20 years to stimulate gross motor and/or locomotor development in infants with disabilities or at risk for developmental delay and to appreciate the characteristics of interventions that appear to enhance an intervention's efficacy. Interventions started as early as possible, with standardized training protocols that encourage active movement, and frequent and focused training sessions appear to be particularly efficacious in promoting gross motor and/or locomotor development. Although the number of studies identified for the review was small, highlighting how difficult and time-consuming early intervention studies are to conduct, our findings reveal that such studies are feasible with a range of different populations of infants, and clearly there is a need for more of them. We urge researchers to continue the difficult work of conducting early gross motor and locomotor interventions and to follow the recommendations we make in the paper to establish proofs of concept that will allow clinicians to confidently adopt the interventions that are most likely to work for their patients.

## Data Availability Statement

The original contributions presented in the study are included in the article/Supplementary Material, further inquiries can be directed to the corresponding author.

## Author Contributions

MD-V and JP searched the databases and summarized their findings on a spreadsheet, including assessment of methodological quality and risk of bias. MB-R and DA independently read the articles for which the full text was to be evaluated. If there was disagreement or discrepancy between the scores, the details were discussed until consensus was reached (MD-V, JP, and MB-R). The summaries are reported in Tables 1–3. The evaluation began with a cross-sectional analysis of the different protocols. The contents of the studies were summarized on one synthetic table (MD-V and JP), focusing on characteristics of study design, population, age of participants, characteristics of the early intervention protocol (type, frequency, and duration of intervention), interveners, assessment, blind assessors and tools used for follow-up. Authors contributed equally to introduction and discussion (MD-V, JP, DA, and MB-R). English was proofread by DA, a native English speaker. All authors contributed to the article and approved the submitted version.

## Funding

This work was supported by the grant ANR-20-CE17-0014 from the Agence Nationale de la Recherche and a fellowship (ED261) from the French Government to support MD-V doctoral thesis.

## Conflict of Interest

The authors declare that the research was conducted in the absence of any commercial or financial relationships that could be construed as a potential conflict of interest.

## Publisher's Note

All claims expressed in this article are solely those of the authors and do not necessarily represent those of their affiliated organizations, or those of the publisher, the editors and the reviewers. Any product that may be evaluated in this article, or claim that may be made by its manufacturer, is not guaranteed or endorsed by the publisher.

## Footnotes

^1^For example, walking parameters such as step length were studied (24), but also, walking cadence, stance phase of the walking cycle and foot roll (25); physical activity rate (26); obstacle avoidance strategies (27); and joint kinematics (28).

^2^I*nternational classification of functioning, disability and health: children and youth version*: ICF-CY. World Health Organization (2007). https://apps.who.int/iris/handle/10665/43737.
