# Supplementary material for: Effects of Early Motor Interventions on Gross Motor and Locomotor Development for Infants at-Risk of Motor Delay: A Systematic Review
Source: Front Pediatr. 2022 Apr 28;10:877345. doi: 10.3389/fped.2022.877345 (PMC9096078; doi:10.3389/fped.2022.877345)
Supplement: Supplementary file 1 [file Data_Sheet_1.pdf]

Appendix S1: Method

Search strategy

A literature search was performed to identify studies published from January 2000 to March 2020. The electronic databases used in this review were PubMed, Embase, PEDRO, Cochrane and WEB of science. The following keywords was used: *Infants* AND *Motor* AND *Training* AND *Therapy*. Publications were included if the groups studied consisted of infants at-risk of motor delay with a prospective or retrospective design. Onset of intervention has to occur before 12 months corrected age for individual infants. Study must include either an experimental group and a comparison group (contemporary or retrospectively) or an experimental period and a comparison period. The effect of intervention on gross motor and/or locomotor development has to be reported. Articles were excluded if the study evaluated multidisciplinary training, pharmaceutical or surgical interventions in addition to motor training, the paper was not written in French or in English to avoid misunderstanding, and the article was a review, unpublished or a published protocol. Interventions that involve a specific motor skill other than locomotion are also excluded (touch, head control, head rotation, fine motor skills).

Evaluation procedure

Two persons (MVD & JP) searched the databases and two reviewers (MBR, DIA) independently read the articles for which the full text was to be evaluated. MVD and JP summarized their findings on a spreadsheet, including assessment of methodological quality and risk of bias. If there was disagreement or discrepancy between the scores, the details were discussed until consensus was reached (MVD, JP, MBR). The summaries are reported in Tables I to III. The evaluation began with a cross-sectional analysis of the different protocols. The contents of the studies were summarized on one synthetic table (MVD, JP), focusing on

characteristics of study design, population, age of participants, characteristics of the early intervention protocol (type, frequency, and duration of intervention), interveners, assessment, blind assessors and tools used for follow-up.

Assessment of the level of evidence according to Sackett et al.(1) and an evaluation of the methodology criteria of the Academy for Cerebral Palsy and Developmental Medicine (AACPDM) for group design studies (revision 1.2, 2008 version) (2) was then performed. To avoid item ambiguity of AACPDM methodology criteria's, footnotes added by Hadders-Algra et al.2017(3) to complement original items, were used. Each item was scored "1" if it was respected and "0" if he was not, resulting in a total score for protocol methodological quality. In accordance with the AACPDM, methodology scores were interpreted as *strong* (6 or 7), *moderate* (4 or 5), or *weak* (≤ 3) methodological quality.

We further analysed the methodology by using the criteria and the Mallen score (Mallen et al.)(4). Twenty-five out of the 30 Mallen criteria were selected, since the last five criteria did not apply to the studies collected in this review. Again, we kept added information by Hadders-Algra et al.2017 (3) to some of the criteria to specify the item. Scoring was still as "1" for a check criterion and "0" it he is not. Total score resulted in the Mallen score (4) (Table SI, online supporting information). Lastly, risk of bias by applying the Cochrane Risk of Bias assessment (5) has been assessed. Authors contributed equally to introduction and discussion (MVD, JP, DIA, MBR). English was proofread by DIA, a native English speaker. The methodological evaluation protocol follows the PICO approach (Participants, Interventions, Comparators, Outcomes) in line with the PRISMA-P statement for reporting systematic reviews (6) and AMSTAR-2. Preregistration was made on PROSPERO database (CRD42021286445).

REFERENCES

1. Sackett DL, Straus SE, Richardson WS, Rosenberg W, Haynes RB. Evidence-Based Medicine: How to Practice and Teach EBM. 2nd Editio. Churchill Livingstone; 2000.

2. Darrah J, Hickman Pt R, O ’donnell M, Msc M, Vogtle L, Wiart L. AACPDM Methodology to Develop Systematic Reviews of Treatment Interventions (Revision 1.2) 2008 Version The Step-by-Step Process for Authors Creating an AACPDM Systematic Review. Milwaukee, WI, USA: American. 2008. 1–30 p.

3. Hadders-Algra M, Boxum AG, Hielkema T, Hamer EG. Effect of early intervention in infants at very high risk of cerebral palsy: a systematic review. Dev Med Child Neurol. 2017;59(3):246–58.

4. Mallen C, Peat G, Croft P. Quality assessment of observational studies is not commonplace in systematic reviews. J Clin Epidemiol. 2006;59(8):765–9.

5. Higgins J, Thomas J, Chandler J, Cumpston M, Li T, Page M, et al. Cochrane Handbook for Systematic Reviews of Interventions [Internet]. 6.2. Higgins J, Thomas J, Chandler J, Cumpston M, Li T, Page M, et al., editors. Cochrane; 2021. Available from: www.training.cochrane.org/handbook

6. Moher D, Shamseer L, Clarke M, Ghersi D, Liberati A, Petticrew M, et al. Preferred reporting items for systematic review and meta-analysis protocols (prisma-p) 2015 statement. Syst Rev. 2015;4(1):1177–85.
