# Supplementary material for: Effects of Early Motor Interventions on Gross Motor and Locomotor Development for Infants at-Risk of Motor Delay: A Systematic Review
Source: Front Pediatr. 2022 Apr 28;10:877345. doi: 10.3389/fped.2022.877345 (PMC9096078; doi:10.3389/fped.2022.877345)
Supplement: Supplementary file 2 [file Data_Sheet_2.pdf]

**Appendix S2**: Results

## Study selection

Figure 1 shows the selection of the articles. The database searches yielded 1235 articles, of which 1129 were excluded on the basis of screening of title and abstract. We assessed the full text of the remaining 106 papers. Eighty-eight were excluded because they did not meet our inclusion criteria, most (n=36) because the interventions were multidisciplinary and not only on motor development. Fifteen excluded articles focused on upper limb therapy and four on head control and/or head rotation. Five articles were a described protocol, four articles were on older children, three articles were published before 2000 and three more articles were case-studies. Finally, eighteen systematic reviews were analyzed on their bibliography to avoid missing an article, but no other articles were added as a result of this review method.

Eighteen articles were eligible in qualitative synthesis, including papers reporting on the same study. Two articles were excluded as one provide insufficient training frequency (a few minutes twice a month) and one had methodological bias on start age for training. Finally, to avoid repetition and to focus on our systematic review goal (motor development and walking acquisition), only original papers were kept (1–3) so six articles reporting on same studies but adding supplementary data were excluded (4–9).

The remaining 10 articles were reviewed in detail. First the studies' methodological quality according to the AACPDM was assessed (10). Then, Mallen score (11) and the risk of bias (12) were assessed and the effect of intervention evaluated.

**Methodological quality**

Three of the 10 studies were small RCTs, implying a level II of evidence according to Sackett (13). One study had a randomized trial with retrospective data as control group. Three studies were not randomized. The three remaining studies were cohort studies without control group, implying a level V of evidence according to Sackett.

The evaluation of methodological quality on the basis of the AACPDM criteria revealed that among the 10 studies, seven had a moderate to strong methodological quality and three studies had a weak methodological quality (Table I). The ten studies all met the criterion 1 (clear description of inclusion and exclusion criteria). In contrast, only three studies met criterion 2 (description of study and control condition including adherence). Criterion 3 (clear description of measures) was met in six studies, four studies met criterion 4 ("Was it explicitly described that the assessors were masked?"). Seven studies met criterion 5 (appropriate statistical evaluation including power calculation) and six studies fulfilled criterion 6 (description of dropouts and limited dropout) whereas five studies met criterion 7 (control for confounding and bias).

The Mallen scores of the ten studies varied from 13 to 22, with a median value of 19,5 (Table SI). The Mallen score did not seem to be related to the year of publication of the papers. Criteria that were fulfilled by all studies were: 'clear inclusion/exclusion criteria', 'clear hypothesis', 'intervention described', 'type of study state', 'main findings described' 'appropriate statistical tests used', 'outcomes clearly described'. Nine studies fulfilled criteria 'disclosure of funding source' and 'conclusion supported by findings'. Eight studies fulfilled criteria 'numerical description of important outcomes given'. Seven studies fulfilled criteria 'potential confounders described', 'appropriate follow-up period (≥ 1 yr)', 'clear case/control definition', 'power

calculation', 'reliable assessment of disease state' and 'reported probability characteristics'. Six studies fulfilled criteria: 'accurate and appropriate outcome measures in all participants available for follow-up', 'adjustment for confounding', 'losses and completers described'. Five studies met criteria 'cases and controls from the same population', 'loss to final follow-up (appropriate level, i.e., <20%)', 'participants representative of population', 'recruitment of case/control over same time frame' and 'participants' characteristics described'. Only four studies met criteria 'blinding of assessors' (Table SI).

The risk of bias in the studies were heterogeneous (Table II). The risk of performance bias and detection bias were mostly unclear from the papers. Selection bias was difficult to evaluate as random sequence generation and allocation concealment were not detailed. Most of the studies were considered to have high risk of performance bias, as families and professionals providing the intervention were aware of its type. Detection bias were also quite important since only four studies had blind assessors. Six studies suffered from attrition bias, most because attrition rate were not presented or analysed. On the contrary, risk of reporting bias was only in two studies. Other risks of bias can be found, for example concerning the assessment of the disease state or whether participants were representative of the general population (see Table SI).

**REFERENCES**

1. Ulrich DA, Ulrich BD, Angulo-Kinzler RM, Yun J. Treadmill training of infants with Down syndrome: evidence-based developmental outcomes. Pediatrics [Internet]. 2001 Nov;108(5):E84. Available from: http://www.embase.com/search/results?subaction=viewrecord&from=export&id=L33545762

2. Ulrich DA, Lloyd MC, Tiernan CW, Looper JE, Angulo-Barroso RM. Effects of intensity of treadmill training on developmental outcomes and stepping in infants with Down syndrome: A randomized trail. Phys Ther [Internet]. 2008 Jan;88(1):114–22. Available from: http://www.embase.com/search/results?subaction=viewrecord&from=export&id=L351020089
3. Campbell S, D G-S, L Z, A C, Boynewicz K, DeRegnier R, et al. Effects on Motor Development of Kicking and Stepping Exercise in Preterm Infants with Periventricular Brain Injury: A Pilot Study. J Pediatr Rehabil Med. 2012;5(1):15–27.
4. Wu J, Looper J, Ulrich BD, Ulrich DA, Angulo-Barroso RM. Exploring effects of different treadmill interventions on walking onset and gait patterns in infants with Down syndrome. Dev Med Child Neurol. 2007 Nov;49(11):839–45.
5. Angulo-Barroso RM, Wu J, Ulrich DA. Long-term effect of different treadmill interventions on gait development in new walkers with Down syndrome. Gait Posture. 2008 Feb;27(2):231–8.
6. Angulo-Barroso R, Burghardt AR, Lloyd M, Ulrich DA. Physical activity in infants with Down syndrome receiving a treadmill intervention. Infant Behav Dev 2008 Apr;31(2)255-269 [Internet]. 2008 Apr;31(2):255–69. Available from: http://www.embase.com/search/results?subaction=viewrecord&from=export&id=L351372864
7. Wu J, Ulrich DA, Looper J, Tiernan CW, Angulo-Barroso RM. Strategy adoption and locomotor adjustment in obstacle clearance of newly walking toddlers with Down syndrome after different treadmill interventions. Exp brain Res. 2007 Mar;186(2):261–72.
8. Wu J, Looper J, Ulrich DA, Angulo-Barroso RM. Effects of Various Treadmill

Interventions on the Development of Joint Kinematics in Infants With Down Syndrome. Phys Ther. 2010;90(9):1265–76.

9. Campbell SK, Cole W, Boynewicz K, Zawacki LA, Clark A, Gaebler-Spira D, et al. Behavior During Tethered Kicking in Infants With Periventricular Brain Injury. Pediatr Phys Ther [Internet]. 2015;27(4):403–12. Available from: http://www.embase.com/search/results?subaction=viewrecord&from=export&id=L615160092

10. Darrah J, Hickman Pt R, O ’donnell M, Msc M, Vogtle L, Wiart L. AACPDM Methodology to Develop Systematic Reviews of Treatment Interventions (Revision 1.2) 2008 Version The Step-by-Step Process for Authors Creating an AACPDM Systematic Review. Milwaukee, WI, USA: American. 2008. 1–30 p.

11. Mallen C, Peat G, Croft P. Quality assessment of observational studies is not commonplace in systematic reviews. J Clin Epidemiol. 2006;59(8):765–9.

12. Higgins J, Thomas J, Chandler J, Cumpston M, Li T, Page M, et al. Cochrane Handbook for Systematic Reviews of Interventions [Internet]. 6.2. Higgins J, Thomas J, Chandler J, Cumpston M, Li T, Page M, et al., editors. Cochrane; 2021. Available from: www.training.cochrane.org/handbook

13. Sackett DL, Straus SE, Richardson WS, Rosenberg W, Haynes RB. Evidence-Based Medicine: How to Practice and Teach EBM. 2nd Editio. Churchill Livingstone; 2000.
