# Supplementary material for: Effects of Early Motor Interventions on Gross Motor and Locomotor Development for Infants at-Risk of Motor Delay: A Systematic Review
Source: Front Pediatr. 2022 Apr 28;10:877345. doi: 10.3389/fped.2022.877345 (PMC9096078; doi:10.3389/fped.2022.877345)
Supplement: Supplementary file 4 [file Data_Sheet_4.pdf]

Appendix S4: Suggestions for future research

Study design

- Precisely define the target population for motor training, in terms of age and pathology.
- If the target population is a population at risk, then precisely define risk and how it is assessed.
- The definition of risk for motor impairment should be constructed taking into account the child's birth term and clinical history.
- Consider the General Movement Assessment and Amiel-Tison's Neurological Assessment as tools to detect at-risk infants.
- Adapt the training to the motor skills of the subjects, so that it is as effective as possible.

Experimental and control group

- In the case of research on brain damaged subjects, the lesions observed in neuroimaging must be matched between the trained group and the control group.
- Define a control group comparable in morphological, clinical and motor skills characteristics.
- A typically-developing group cannot consist of children born prematurely because premature birth induces a risk of developmental delay.
- Group size should be adapted to obtain sufficient statistical power.

Training design

- In order to stimulate motor development, start motor training as early as possible to take advantage of the highest potential for brain and body plasticity.
- The frequency of the training determines the choice of the person who will carry out the sessions: daily training involves the parents; training once or twice a week can be done by a therapist.
- The duration of the training must be considered in relation to the planned analyses: if it is adapted to the subject, this will lead to an analysis by subject in relation to time 0 and then possibly a comparison of the progression between subjects; if the duration is fixed, then a comparison between subjects will be possible at any time during the follow-up.
- The training modality must be the same within the trained group, for example a manual therapy protocol must be explicitly detailed and followed by all the therapists in order to conclude that the training has had an effect.
- The actual duration of the sessions must be recorded, if possible, by a sensor that cannot be modified by the parents, which also makes it easier for the parents to follow the protocol and avoids them having to fill in a logbook or make videos (and analyze them).
- The duration of the longitudinal follow-up must be determined in relation to the objective of the motor training.

Intervention description

- Describe details of the contents of the experimental and control intervention, including frequency and duration of intervention sessions, location of intervention, and professionals delivering the intervention service.
- Evaluation of behavioural state (Prechtl 1974) is a prerequisite to assessing and training an infant.
- For motor training, the active production of movement by the subject can be likened to operant conditioning, stimulating the child's motor production with positive reinforcement to elicit voluntary motor skills. The child who is proactive during training will better retain the movements learned during training.
- Motor training has to be well-tolerated by infant's family.
- Consider an ICF-CY approach including the ability to act on one's environment as an important reward; all systems should promote autonomy; maximize development of motor potential for each subject; specialize therapy and use of assistive devices when its needed.

Assessment methodology

- A multidisciplinary approach can reveal a multifactorial effect but cannot prove a specific effect of one of the components. To prove the efficacy of a specific motor training component, ensure that component can be assessed in isolation even if the intervention is multidisciplinary.
- Report details on birth characteristics and morphological data for each group, in order to compare motor development. If potential confounders appear, covariance analysis is necessary.
- Perform evaluation by blind assessors.
- To evaluate the effects of motor training, assessments at a minimum of three time points are needed: baseline, immediately after training and following a delay after training.
- Standardized tools are necessary for replication and comparison of results.
- Standardized tools must be concordant with training objectives.
- Parents' participation is one essential aspect of a successful training because it helps to avoid attrition.
- Studying infants at-risk of motor delay involves taking into consideration parental psychological impact; longitudinal studies must be adapted to limit parental overload.
- Attrition number and reason for attrition must be mentioned to avoid attrition bias on the results and to avoid replication of unsuccessful studies.
- Special attention is needed to the normalization of context for assessment and home-training.
- Written recommendations for parents to oversee training include doing training in one dedicated place without other children, animals or distraction, and conducting the session when the infant is calm but awake and not immediately after a meal.
