# Supplementary material for: Effects of Early Motor Interventions on Gross Motor and Locomotor Development for Infants at-Risk of Motor Delay: A Systematic Review
Source: Front Pediatr. 2022 Apr 28;10:877345. doi: 10.3389/fped.2022.877345 (PMC9096078; doi:10.3389/fped.2022.877345)
Supplement: Supplementary file 5 [file Table_1.pdf]

Table SI: Mallen scores: extended methodological quality assessment

Mallen quality criteria	Ulrich et al.2001	Ulrich et al.2008	Angulo-Barrosso et al.2013	Campbell et al. 2012	Lee & Samson 2019	Schlitter et al. 2011	Kolobe & Fagg 2019	Wentz 2017	Cameron et al. 2005	Ustad et al.2009
Accurate and appropriate outcome measures in all participants *available for follow-up*[a]	1	1	1	0	1	0	1	1	0	0
Adjustment for confounding *by multivariable statistic or subgroup analysis*[a]	1	1	1	0	1	0	0	1	1	0
Cases and controls from same population[a]	1	1	0	1	0	0	0	1	1	0
Loss to *final* follow-up *(appropriate level, i.e. <20%)*[a]	0	0	1	1	1	0	0	1	1	0
Appropriate statistical tests used, and tests described	1	1	1	1	1	1	1	1	1	1
Participants representative of population[a]	1	1	0	1	0	0	0	1	1	0
Potential confounders described[a]	1	1	1	1	1	0	0	0	1	1
Recruitment of case/control over same time frame (or similar point of disease)[a]	1	0	1	1	0	1	0	0	1	0
Blinding (assessors)[a]	0	0	0	1	0	0	1	0	1	1
Participants characteristics described[b]	1	1	1	1	0	0	0	0	1	0

Table SI continued: Mallen scores: extended methodological quality assessment

Mallen quality criteria	Ulrich et al.2001	Ulrich et al.2008	Angulo-Barrosso et al.2013	Campbell et al. 2012	Lee & Samson 2019	Schlitter et al. 2011	Kolobe & Fagg 2019	Wentz 2017	Cameron et al. 2005	Ustad et al.2009
Numerical description of important outcomes given	1	1	1	1	0	0	1	1	1	1
Outcomes clearly described[a]	1	1	1	1	1	1	1	1	1	1
Appropriate follow-up period (≥ 1 yr)	1	1	1	1	1	1	0	1	0	0
Clear case/control definition[a]	1	1	0	1	0	1	1	1	1	0
Power calculation used	1	1	1	0	0	1	1	1	1	0
Losses and completers described[a]	0	1	1	1	1	1	0	0	1	0
Reliable assessment of disease state[a,c]	1	1	0	1	1	0	1	0	1	1
Clear inclusion/exclusion criteria[a]	1	1	1	1	1	1	1	1	1	1
Clear hypothesis[a,d]	1	1	1	1	1	1	1	1	1	1
Intervention described[a]	1	1	1	1	1	1	1	1	1	1
Reported probability characteristics	1	1	1	1	0	0	1	1	1	0
Type of study stated	1	1	1	1	1	1	1	1	1	1
Main findings described[a]	1	1	1	1	1	1	1	1	1	1
Disclosure of funding source[a]	1	1	1	1	1	1	1	1	0	1

Conclusion supported by findings[e]	1	1	1	1	1	0	1	1	1	1
**Total score (max 25)**	**22**	**22**	**20**	**22**	**16**	**13**	**16**	**19**	**22**	**13**

Adapted Mallen score[28]: the highest possible score is 25. 1=yes; 0=no. If the paper did not provide information needed for a criterion a 'no'-score was assigned. * determined on the basis of additional information provided by the authors.

[a] Criterion indicating risk of bias

[b] At least description of 1) frequencies of specific brain lesions or prevalence of CP; 2) gestational age at birth or proportion of preterm infants; 3) birth weight or proportion of infants with low birth weight; the three criteria need to be met in order to score 'yes'.

[c] Brain lesions: criteria should be specified, either in the text or with appropriate reference; for a diagnosis of CP infants should be at least ≥ 12 mo CA.

[d] Clear hypothesis means that the paper specifies with a hypothesis or specific research question which effects of intervention are expected, by specifying the type of effect of the intervention.

[e] The criterion is not met if the conclusion summarises findings correctly, e.g., reports that no statistically significant differences between study and control group were found, but nevertheless concludes that the study supports a favourable effect of the intervention.
